# Predictors of futile recanalization in nonagenarians treated with mechanical thrombectomy: a multi-center observational study

**DOI:** 10.1007/s00415-024-12428-8

**Published:** 2024-05-16

**Authors:** Lucio D’Anna, Giovanni Merlino, Michele Romoli, Liqun Zhang, Caterina Del Regno, Mohammed Aggour, Viva Levee, Matteo Foschi, Massimo Sponza, Francesco Toraldo, Razan Algazlan, Maria Ruggiero, Marco Longoni, Kyriakos Lobotesis, Samir Abu-Rumeileh, Daniele Bagatto, Nina Mansoor, Gian Luigi Gigli, Mariarosaria Valente, Soma Banerjee

**Affiliations:** 1 Department of Stroke and Neuroscience, Charing Cross Hospital, Imperial College London NHS Healthcare Trust, London, UK; 2 Department of Brain Sciences, Imperial College London, London, UK; 3 Stroke Unit, Udine University Hospital, Udine, Italy; 4 Clinical Neurology, Udine University Hospital and DAME, University of Udine, Udine, Italy; 5 Stroke Unit and Clinical Neurology, Udine University Hospital, Udine, Italy; 6 Neurology and Stroke Unit, Department of Neuroscience, Bufalini Hospital, AUSL Romagna, Cesena, Italy; 7 Department of Neuroscience, George’s University of London, Stroke, London, UK; 8 Department of Biotechnological and Applied Clinical Sciences, University of L’Aquila, L’Aquila, Italy; 9 Neuroradiology, Udine University Hospital, Udine, Italy; 10 Neuroradiology, Bufalini Hospital, AUSL Romagna, Cesena, Italy; 11 Neuroradiology, Department of Imaging, Charing Cross Hospital, Imperial College London, NHS Healthcare Trust, London, UK; 12 Department of Neurology, Martin-Luther-University Halle-Wittenberg, Halle (Saale), Germany

**Keywords:** Futile recanalization, Nonagenarians, Intravenous thrombolysis, Mechanical thrombectomy

## Abstract

**Background:**

There is a lack of data regarding patients aged 90 years or older undergoing mechanical thrombectomy and their predictors of futile recanalization.

**Aims:**

We sought to evaluate the predictors of futile recanalization in patients ≥ 90 years with large vessel occlusion undergoing mechanical thrombectomy.

**Methods:**

This multi-center observational retrospective study included patients ≥ 90 years consecutively treated with mechanical thrombectomy in four thrombectomy capable centers between January 1st, 2016 and 30th March 2023. Futile recanalization was defined as large vessel occlusion patients experiencing a 90-day poor outcome (mRS 3–6) despite successful recanalization (mTICI ≥ 2b) after mechanical thrombectomy.

**Results:**

Our cohort included 139 patients ≥ 90 years with acute ischemic stroke due to anterior circulation large vessel occlusion treated with mechanical thrombectomy. One hundred seventeen of one hundred thirty-nine patients ≥ 90 years who achieved successful recanalization were included in the analysis (seventy-six female (64.9%)), of whom thirty-one (26.49%) experienced effective recanalization and eighty-six (73.51%) experienced futile recanalization. Patients with futile recanalization had higher NIHSS on admission (*p* < 0.001); they were less frequently treated with intravenous thrombolysis (*p* = 0.048), had more often general anesthesia (*p* = 0.011), and longer door to groin puncture delay (*p* = 0.002). Univariable regression analysis showed that use of intravenous thrombolysis (0.29, 95% CI 0.02–0.79, *p* = 0.034) and site of occlusion distal vs proximal (0.34, 95% CI 0.11–0.97, *p* = 0.044) were associated with reduced probability of futile recanalization while NIHSS on admission (1.29, 95% CI 1.16–1.45, *p* < 0.001), NIHSS at 24 h (1.15, 95% CI 1.07–1.25, *p* = 0.002), type of anesthesia used (4.18, 95% CI 1.57–11.08, *p* = 0.004), and door to groin puncture time (1.02, 95% CI 1.00–1.05, *p* = 0.005) were associated with increased probability of futile recanalization. Multivariable regression analysis showed that use of intravenous thrombolysis (0.44, 95% CI 0.09–0.88, *p* = 0.039) was associated with reduced probability of futile recanalization.

**Conclusion:**

Our study seems to suggest that mechanical thrombectomy with intravenous thrombolysis is associated with reduced probability of futile recanalization in a multi-center cohort of patients aged 90 years or older.

**Supplementary Information:**

The online version contains supplementary material available at 10.1007/s00415-024-12428-8.

## Introduction

Mechanical thrombectomy (MT) currently represents the standard of care of acute ischemic stroke due to large vessel occlusion (LVO) regardless of the age of the patient [1]. A recent analysis of data from the Multicenter Randomized Clinical Trial of Endovascular Treatment for Acute Ischemic Stroke (MR CLEAN) including 2807 patients with a median age ranging from 71 to 75 years reported the association between clinical outcome and the thrombolysis in cerebral infarction (TICI) classification scores [2]. Functional independence at 90 days from the index event was achieved in 42.8% of TICI 2B, 49.3% of TICI 2C, and 51.9% of TICI 3 patients. Although major clinical trials have demonstrated the benefit of MT for the general population, the degree of benefit is less clear in very elderly patients aged 90 years or older as this subgroup was often excluded or under-represented in past trials [3–6]. Indeed, although previous observational studies have shown that a good functional outcome is achieved in a low rate of nonagenarians who underwent MT following an acute ischemic stroke, MT in nonagenarians is associated with a recanalization rate often comparable to younger patients [7–12]. Therefore, there is still a significant proportion of patients aged ≥ 90 who do not achieve a good functional outcome at 90 days after MT despite successful recanalization, also known as futile recanalization.

Previous studies have investigated predictors of futile recanalization of MT in acute ischemic stroke caused by LVO in the anterior circulation for the general population [13, 14]. However, there is a lack of data specifically regarding patients aged 90 years or older and their predictors of futile recanalization after MT. Considering the overall aging population, it is expected that an increasing number of patients aged 90 years or older with LVO will meet the criteria for MT in the future. Therefore, it is of paramount importance to better understand the efficacy of MT in this age group and to define factors that might help to identify patients likely to benefit from the treatment. We conducted a multi-center observational retrospective study aiming to explore the rate and predictors of futile recanalization in patients aged 90 years or older with LVO undergoing MT.

## Methods

### Study design and patients

This is a multi-center, observational, investigator-initiated, retrospective study, that included all acute stroke patients aged 90 years or older consecutively treated with MT in four thrombectomy capable centers: Charing Cross Hospital, Imperial College Healthcare NHS Trust, London (UK); St George’s University of London, London (UK); Udine University Hospital, Udine (Italy); Bufalini Hospital, AUSL Romagna, Cesena, (Italy) between January 1st, 2016 and 30th March 2023 with local stroke registries available [15–18]. The study was conducted in accordance with the recommendations for physicians involved in research on human subjects adopted by the 18th World Medical Assembly, Helsinki 1964 and later revisions.

### Patient inclusion and exclusion criteria for the analysis

For the purpose of this analysis, the criteria for patients selection were: (1) age ≥ 90 years; (2) National Institutes of Health Stroke Scale (NIHSS) score 6 or more; (3) Alberta Stroke Program Early CT score (ASPECTS) 5 or more [19]; (4) LVO sites: distal internal carotid artery, middle cerebral artery segments M1 or M2; (5) initiation of the MT had to be possible within 6 h after the stroke onset; (6) pre-event modified Rankin Scale (mRS) score of 0 to 2. Intravenous thrombolysis (IVT) with intravenous tissue plasminogen activator (tPA) was administered in all patients who presented within 4.5 h of stroke symptom onset and without contraindications according to the guidelines. For this analysis, we excluded stroke patients with basilar artery occlusion and patients that met DAWN or DEFUSE 3 eligibility criteria [20, 21].

### Data collection

All information were collected prospectively, such as medical history, demographic characteristics, history of previous stroke or transient ischemic attack (TIA), baseline NIHSS, admission therapy, site of the occlusion, procedural management and variables, key time points. The modified Rankin Scale (mRS) was used to assess the patient’s initial pre-stroke status and at 90 days. Two independent and trained raters for each center who did not participate in the endovascular stroke treatment of the included patients and blind to any clinical and treatment information evaluated the modified Rankin Scale (mRS) of the patients at 90 days centrally through a telemedicine consultation or in-person consultation. Any disagreement was resolved with the involvement of a senior stroke neurologist for each center as third party not involved in the care of the patients. The investigators received training and qualification certificates to record NIHSS and mRS. NIHSS was performed in all patients on admission and 24 h after the MT. The prescription of any antiplatelets and anticoagulant before admission was recorded and included the use of any direct oral anticoagulant (DOAC) therapy (defined as one of the following drugs and dosages: apixaban 2.5 mg or 5 mg twice daily; dabigatran 110 mg or 150 mg twice daily; edoxaban 30 mg or 60 mg once daily; or rivaroxaban 15 mg or 20 mg once daily).; vitamin K antagonist (VKA) (defined as treatment with acenocoumarol/ warfarin). The choice of treatment was decided by the treating physician as part of routine clinical care pre-admission. The extent of the initial core infarct was determined on pre-therapeutic CT using ASPECTS. In addition, independent raters (consultant neuroradiologists) who did not participate in the endovascular stroke treatment of included patients evaluated pre-therapeutic CT and follow-up CT at 24 h. Revascularization was assessed by applying the modified thrombolysis in cerebral infarction (TICI) classification [22]. Successful recanalization was defined as grade 2b, 2c or 3 of recanalization. The pre-therapeutic CT was evaluated to assess the collateral score (CS). CS grading was based on the five-point grading system proposed by Souza et al [23]. Intracranial CTA maximum intensity projections were used for grading the CS: 0 = absent collaterals in > 50% of an MCA M2 branch (superior or inferior division) territory; 1 = diminished collaterals in > 50% of an MCA M2 branch territory; 2 = diminished collaterals in < 50% of an MCA M2 branch territory; 3 = collaterals equal to the contralateral hemisphere; and 4 = increased collaterals.

Hemorrhagic transformation was defined as symptomatic intracerebral hemorrhage (sICH) if it was not seen on the admission brain scan and there was, subsequently, a suspicion of hemorrhage or a decline in neurological status (an increase of more than 4 points in the NIHSS) (sICH; according to Safe Implementation of Thrombolysis in Stroke-Monitoring Study [SITS-MOST], European Cooperative Acute Stroke Study-II [ECASS-II], Solitaire With the Intention for Thrombectomy as Primary Endovascular Treatment [SWIFT- PRIME]).

Anesthesia was provided by neuroanesthesists who were present for all MT procedures as general anesthesia (GA) or local anesthesia (LA). For the purpose of this analysis, we considered GA patients those who initially underwent LA and then subsequently converted to GA.

### Futile recanalization and effective recanalization

We defined futile recanalization as LVO patients experiencing a 90-day poor outcome (mRS 3–6) despite successful recanalization (mTICI ≥ 2b) after MT and effective recanalization as LVO patients achieving a 90-day good outcome (mRS ≤ 2) with successful recanalization after MT.

### Statistical analysis

Descriptive categorical data were reported as numbers and proportions; descriptive continuous data were reported as means and standard deviations (SDs) for normally distributed variables, or medians and interquartile ranges (IQRs) for non-normally distributed variables. We compared the demographic, clinical, and procedure-related characteristics of the two groups (effective recanalization vs futile recanalization) by Chi-square test (for categorical variables), one-way ANOVA (for normally distributed continuous variables, followed by Tukey’s post hoc test), or Kruskal–Wallis test (for non-normally distributed continuous variables). *p* values were considered statistically significant at < 0.10. We performed a univariate logistic regression analysis with calculation of odds ratios (ORs) and 95% confidence intervals (CIs) to investigate variables associated with the study outcomes. Variables with a significant association with the study outcomes (*p* ≤ 0.05) were considered for multivariate logistic regression analysis with statistical significance set at a *p* < 0.05. Statistical analyses were performed with R software, version 4.2.2.

## Results

**Fig. 1:**
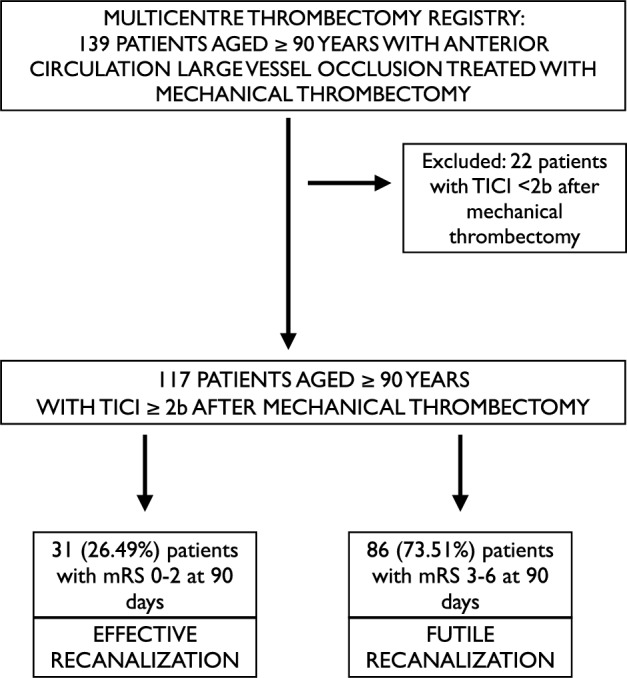
Study flow chart

Our cohort included 139 patients aged 90 years or older with acute ischemic stroke due to anterior circulation LVO consecutively treated with MT in four thrombectomy capable centers. One hundred seventeen of one hundred thirty-nine patients aged 90 years or older achieved successful recanalization after MT and were included in the analysis, of whom thirty-one (26.49%) experienced effective recanalization and eighty-six (73.51%) experienced futile recanalization (see flow chart Fig. 1).

The demographical and clinical data of the patients with effective and futile recanalization are presented in Table 1. Overall, the mean age was 92 years (IQR 90–93) and 64.9% were female (*n* = 76). The median ages in the two groups did not differ significantly. The two groups did not differ significantly for most of the variables taken into account except for the fact that patients with futile recanalization had a higher NIHSS on admission (*p* < 0.001).

**Table 1:** Demographic and clinical characteristics

	Overall population (> 90) TICI 2b,2c,3 (*N* = 117)	Effective recanalization (*N* = 31)	Futile recanalization (*N* = 86)	*p*
*Demographics*				
Age, years [median (IQR)]	92 (90–93)	92 (90–92.5)	92 (90–93)	0.747
Female sex [*n*, (%)]	76 (64.9)	21 (67.7)	55 (63.9)	0.873
Hypertension [*n*, (%)]	72 (61.5)	20 (64.5)	52 (60.5)	0.497
Diabetes mellitus [*n*, (%)]	11 (9.4)	2 (6.4)	9 (10.4)	0.766
Hypercholesterolemia [*n*, (%)]	45 (38.5)	12 (38.7)	33 (38.4)	0.497
Atrial fibrillation [*n*, (%)]	62 (52.3)	16 (51.6)	46 (53.5)	0.418
Coronary artery disease [*n*, (%)]	10 (8.5)	3 (9.6)	7 (8.1)	1
Congestive heart failure [*n*, (%)]	21 (17.9)	6 (19.4)	15 (17.4)	0.609
Previous TIA/ischemic stroke [*n*, (%)]	11 (9.4)	3 (9.6)	8 (9.3)	1
Dementia [*n*, (%)]	1 (1.4%)	1 (3.2)	–	–
Malignancy [*n*, (%)]	20 (17.1)	5 (16.1)	15(17.4)	0.998
*Pre-mRS* [median (IQR)]	1 (0–2)	1 (0–2)	1 (0–2)	0.859
*Admission therapy*				
Anticoagulation on admission [*n*, (%)]	24 (20.5)	–	24	–
Antiplatelet therapy on admission [*n*, (%)]	20 (17.1)	–	20	–
NIHSS on admission [median (IQR)]	17 (14–20)	12 (10–16)	18 (17–21)	** < 0.001**
ASPECTS score [median (IQR)]	9 (8–10)	9 (9–10)	9 (8–10)	0.097

*mRS*  modified Rankin Scale, *NIHSS*  National Institutes of Health Stroke Scale, *ASPECTS*  The Alberta Stroke Program Early CT Score, *TICI*  modified thrombolysis in cerebral infarction classification, *TIA*  transient ischemic attack

Table 2 shows the comparison of the procedural features between patients with effective and futile recanalization patients aged 90 years or older. The two groups did not differ in terms of site of occlusion, CS, symptom onset to door time, door to needle time, symptom onset to groin puncture time, and rate of sICH. The median ASPECTS score at 24 h was significantly lower in patients with futile recanalization (*p* = 0.004). Patients with futile recanalization were less frequently treated with IVT (*p* = 0.048) and underwent the procedure more often with GA (*p* = 0.011). They also had a higher NIHSS at 24 h from MT (*p* < 0.001) and longer door to groin puncture delay (*p* = 0.002).

**Table 2:** Procedural features

	Overall population (> 90) TICI 2b,2c,3 (*N* = 117)	Effective recanalization (*N* = 31)	Futile recanalization (*N* = 86)	*p*
*Site of occlusion [n, (%)]*				0.059
Distal ICA	15 (12.8)	1 (3.2)	14 (16.3)	
M1	78 (66.7)	19 (61.3)	59 (68.6)	
M2	17 (14.5)	8 (25.8)	9 (10.5)	
ICA + M1	7 (6)	3 (9.6)	4 (4.6)	
*Collateral score*				
0 [*n*,(%)]	26	4 (12.9)	22 (25.6)	0.268
1 [*n*,(%)]	42	14 (45.2)	28 (32.6)	
2 [*n*,(%)]	34	7 (22.6)	27 (31.4)	
3 [*n*,(%)]	9	4 (12.9)	5 (5.8)	
4 [*n*,(%)]	6	2 (6.4)	4 (4.6)	
*IVT [n, (%)]*	84 (71.8)	27 (87.1)	57 (66.3)	**0.048**
*Anesthesia used [n, (%)]*				**0.011**
LA	51 (43.6)	23 (74.2)	28 (32.6)	
GA	66 (56.4)	8 (25.8)	58 (67.4)	
NIHSS 24 h [median (IQR)]	10 (5–17)	4 (1–8)	14 (8–20)	** < 0.001**
ASPECTS score 24 h [median (IQR)]	7 (6–8.75)	8 (7–9)	7 (6–8)	**0.004**
Symptom onset to door time (min), [median (IQR)]	88.5 (73–132.75)	88.5 (79–118)	88.5 (70–141)	0.563
Door to needle time (min), [median (IQR)]	35.5 (26–48)	35 (26–48)	35.5 (26.5–47)	0.727
Symptom onset to groin puncture time (min), [median (IQR)]	245 (197.5–306)	245 (174.5–341)	245 (202.5–295.5)	0.624
Door to groin puncture time (min), [median (IQR)]	111.5 (66–144.75)	91.5 (41–103)	111.5 (96.5–151)	**0.002**
*sICH*	7 (6)	1 (3.2)	6 (6.9)	0.811

*TICI*  modified thrombolysis in cerebral infarction classification, *IVT* intravenous thrombolysis, *NIHSS*  National Institutes of Health Stroke Scale, *sICH* symptomatic intracerebral hemorrhage, *GA*  general anesthesia, *LA*  local anesthesia, *ICA*  internal carotid artery, *M1, M2*  middle cerebral artery segments M1 or M2

As shown in Table 3, after the multivariable logistic analysis, in patients aged 90 years or older with LVO treated with MT, the use of IVT was associated with reduced probability of futile recanalization (0.44, 95% CI 0.09–0.88, *p* = 0.039), while door to groin puncture delay (1.02, 95% 1.001–1.05, *p* = 0.026) was positively correlated with futile recanalization.

**Table 3:** Logistic regression analysis for mRS 3–6 at 90 days in patients ≥ 90 years of age

	Univariate analysis			Multivariate analysis		
	OR (95% CI)	*z*	*p*	OR (95% CI)	*z*	*p*
IVT	0.29 (0.02–0.79)	0.120	**0.034**	0.44 (0.09–0.88)	0.14	**0.039**
NIHSS on admission (per 1 point increase)	1.29 (1.16–1.45)	4.897	** < 0.001**	0.98 (0.79–1.21)	0.763	0.898
NIHSS 24 h (per 1 point increase)	1.15 (1.07–1.25)	4.195	**0.002**	1.15 (1.00–1.32)	7.45	0.045
Anesthesia used (GA vs LA)	4.18 (1.57–11.08)	17.76	**0.004**	2.80 (0.43–18.12)	2.94	0.279
Door to groin puncture time per min	1.02 (1.006–1.05)	17.08	**0.005**	1.02 (1.00–1.05)	9.23	**0.026**
Site of occlusion distal (M2) vs proximal (ICA, M1, ICA + M1)	0.34 (0.11–0.97)	− 2.01	**0.044**	0.61 (0.07–4.90)	0.63	0.643
ASPECTS score (per 1 point increase)	0.57 (0.17–1.88)	− 0.92	0.997	–	–	–
ASPECTS score 24 h (per 1 point increase)	0.63 (0.09–2.70)	0.99	0.993	–	–	–

*TIA*  transient ischemic attack, *IVT*   intravenous thrombolysis, *NIHSS*  National Institutes of Health Stroke Scale, *GA*  general anesthesia, *LA*  local anesthesia

## Discussion

The main finding of our multi-center observational study is that the use of intravenous thrombolysis was associated with a reduced probability of futile recanalization in patients aged 90 years or older with LVO undergoing MT. Current guidelines recommend IVT with alteplase for patients over 80 years of age with acute ischemic stroke within the 4.5-h window [24]. However, data are relatively limited regarding the specific benefits and risks of IVT in nonagenarians, since many clinical trials of tPA did not include patients aged > 80 years, and tPA has strict selection criteria that exclude many of these patients from eligibility. Indeed, Arora et al. documented in their national quality registry that up to one-third of patients ≥ 90 years eligible for lytic therapy did not receive IVT [25]. This was primarily because of fear that advancing age is associated with poorer prognosis with greater risk for symptomatic intracerebral hemorrhage and in-hospital mortality. Moreover, nonagenarian patients have often already a higher burden of morbidity and premorbid baseline disability. It is noteworthy to mention that in our study, all the patients included in the analysis had a baseline mRS score ≤ 2 and this might be one of the reasons to explain our high rate of use of IVT with almost 70% of our nonagenarian patients treated with lytic therapy. Data on bridging thrombolysis in the elderly population remain sparse and equivocal, to date. Although thrombolytic agents, such as alteplase, may contribute to early recanalization, IVT could delay the MT, increase the risk of symptomatic intracerebral hemorrhage, or distal migration of thrombi, rendering them inaccessible to MT. Previous randomized controlled trials have addressed this question, and although some trials met liberal noninferiority end points, noninferiority of MT alone compared with MT with bridging thrombolysis could not be uniformly established [26–29]. In the SKIP trial [26] and SWIFT-DIRECT trial [30], subgroup analyses showed that in patients older than 70 years old, MT with or without bridging IVT led to no differences in outcomes. Retrospective studies evaluated the safety and efficacy of IVT before MT in elderly patients [31–33]. An analysis based on the data of the ANGEL-ACT registry documented that MT alone had similar efficacy to bridging MT in terms of 90-day functional outcomes in 482 patients ≥ 65 years [33]. In this present analysis, the authors found that the time from door to groin puncture was longer in the bridging group which could lead to the conclusion that bridging IVT might delay the time for endovascular treatment. Conversely, Barral et al. showed that bridging IVT was associated with better outcomes in 169 patients with anterior circulation LVO over 80 [31]. In our study, patients with effective recanalization after MT were more often treated with also IVT, and also had a shorter door to groin puncture time compared to patients with futile recanalization. Of note, in our analysis, we documented a strong trend to futile recanalization in patients with proximal occlusion rather than distal. However, while, on univariable analysis, the site of occlusion distal (M2) vs proximal (ICA, M1, ICA + M1) resulted to be significantly associated with decreased likelihood of futile reperfusion, this variable did not result to be significantly associated with reduced probability of futile reperfusion on multivariable analysis although we can appreciate a trend toward this direction. Moreover, in our analysis, door to groin puncture time delay is associated with increased risk of functional dependence at 90 days after the stroke. This finding may be explained by the synergistic action of intravenous thrombolysis and MT in targeting small vessel recanalization and improving distal microvascular perfusion. Future studies are still needed to fully elucidate the efficacy and safety of bridging thrombolysis in nonagenarians.

Another important finding of our study is that 73.51% of the nonagenarian patients experienced futile recanalization after MT and this is in line with previous studies. In the analysis of Drouard-de Rousiers et al., among 124 nonagenarians, treated with MT those with successful recanalization were 93, of whom 18 (19%) experienced effective recanalization while 75 (81%) experienced futile recanalization [34]. Meyer et al. described outcome and safety of a large cohort of patients ≥ 90 years undergoing MT [35]. In this study, the authors found that the frequency of good recanalization (TICI 2b or 3) was not significantly different in patients with good functional outcome compared to those with mRS ≥ 4 at 90 days after the event. Interestingly, in this study, successful recanalization was not a predictor of good outcome after MT. According to the authors, this could be explained by the fact that a significant proportion of patients worsened regardless the successful recanalization. Unfortunately, the authors did not provide data regarding the exact rate and predictors of futile recanalization in their patient sample. Singer et al. investigated the impact of patient age on the outcomes post-MT for anterior circulation LVO [36]. The authors found that the proportion of patients with futile recanalization increased from 29% (age group 18–53 year old) to 53% in patients within the age group 77–94 years old. Overall, the current literature clearly suggested that the effect of recanalization on 90-day outcome seems to be lower in patients ≥ 90 years. The aging process of the cerebral vessels, in terms of atherosclerosis, amyloid angiopathy, friability, small vessel disease [37] (including leukoaraiosis and brain atrophy) may be important factors to determine futile recanalization after MT [38, 39]. In addition, in older patients, the presence of poor collateral circulation, increased risk of hemorrhagic transformation and impaired cerebrovascular autoregulation, and large hypoperfusion volume could also lead to earlier tissue loss and therefore worse functional outcomes than younger patients [40, 41]. Therapeutic strategies targeting tissue recanalization and attenuation of futile recanalization in the clinical setting are needed.

Finally, our study confirmed the correlation between door to groin puncture delay and treatment outcomes. In-hospital delays include any portion of the neurointerventional activation process and, therefore, represent a significant opportunity to improve time to treatment. Interestingly, in our study, we did not observe any significant difference in terms of pre-hospital delay and door to needle time between patients with futile recanalization and effective recanalization. However, we showed that patients with futile recanalization experienced longer door to groin puncture time compared to those with effective recanalization. Based on our findings, we could not clarify the reasons for this significant treatment delay. Nevertheless, it is noteworthy to mention that patients with futile recanalization had a higher NIHSS score on admission and more often had GA as choice of sedation for the procedure. However, in our cohort, the median door to groin time (min) in patients with GA was not significantly higher compared to patients with LA, i.e., respectively, 118 (88.5–143.5) vs 111.5 (59.75–147) (*p* = 0.443). Therefore, it is possible to hypothesize that the increased stroke severity could have impacted on the hyperacute stroke management. Treatment delays not only limit candidacy for MT, but also worsen functional disability and reduce number of healthy life years after treatment [42, 43].

Our analysis had the following strengths: (1) large cohort of nonagenarian patients; (2) multi-center study. Nevertheless, our study has several limitations. First is the non-randomized design of the study that might have introduced bias. Even though we adjusted for this factor in the logistic regression analyses to determine their impact on the outcomes, this could represent a potential bias. Infarct volume was not available. Finally, our analysis included patients who had the initiation of the procedure within 6 h after the stroke onset.

## Conclusion

Our study seems to suggest that MT with IVT is associated with reduced probability of futile recanalization in patients aged 90 years or older. Further studies are warranted to confirm the results and to evaluate the safety and efficacy of bridging thrombolysis in nonagenarians.

### Supplementary Information

Below is the link to the electronic supplementary material.

Supplementary file1 (DOCX 17 KB)

## Data Availability

Data available upon reasonable request.
