# Supplementary material for: Predictors of futile recanalization in nonagenarians treated with mechanical thrombectomy: a multi-center observational study
Source: J Neurol. 2024 May 16;271(8):4925–32. doi: 10.1007/s00415-024-12428-8 (PMC11319431; doi:10.1007/s00415-024-12428-8)
Supplement: Supplementary file 1 — Supplementary file1 (DOCX 17 KB) [file 415_2024_12428_MOESM1_ESM.docx]

**Supplemental Table 1.**

|  | **Patients <90**  **(N=874)** | **Patients ≥90**  **(N=117)** | **p** |
| --- | --- | --- | --- |
| **Demographics** |  |  |  |
| Age, years [median (IQR)] | 69.7 (55-84) | 92 (90-93) | **<0.001** |
| Female sex [n, (%)] | 429 (49.1) | 76 (64.9) | **<0.001** |
| Hypertension [n, (%)] | 346 (39.6) | 72 (61.5) | **<0.001** |
| Diabetes mellitus [n, (%)] | 145 (16.6) | 11 (9.4) | 0.070 |
| Hypercholesterolemia [n,  (%)] | 312 (35.7) | 45 (38.5) | 0.679 |
| Atrial fibrillation [n,  (%)] | 458 (52.4) | 62 (52.3) | 0.900 |
| Coronary artery disease [n, (%)] | 82 (17.7) | 10 (8.5) | **<0.001** |
| Congestive heart failure [n, (%)] | 46 (9.4) | 21 (17.9) | **<0.001** |
| Previous TIA/ischemic stroke [n, (%)] | 97 (11.2) | 11(9.4) | 0.878 |
| Dementia [n, (%)] | - | 1 (1.4%) | - |
| **Pre-mRS** [median (IQR)] | 0 (0-2) | 1 (0-2) | 0.080 |
| **Admission therapy** |  |  |  |
| Anticoagulation on  admission [n, (%)] | 84 (17.1) | 24(20.5) | 0.657 |
| Antiplatelet therapy on  admission [n, (%)] | 196 (28.7) | 20(17.1) | 0.010 |
| NIHSS on admission [median (IQR)] | 17 (12 - 20) | 17 (14-20) | 0.300 |
| ASPECTS score [median (IQR)] | 10 (9 - 10) | 9 (8-10) | 0.432 |
| Symptom onset to door time (min), [median (IQR)] | 85 (71-129.7) | 88.5 (73-132.75) | 0.753 |
| Door to needle time (min), [median (IQR)] | 34.7 (25-47) | 35.5 (26-48) | 0.258 |
| Symptom onset to groin puncture time (min), [median (IQR)] | 245 (195 - 306) | 245  (197.5-306) | 0.987 |
| Door to groin puncture time (min), [median (IQR)] | 110.1 (65-142.9) | 111.5 (66-144.75) | 0.876 |
